# Reduction of prevalence of patients meeting the criteria for metabolic syndrome with tirzepatide: a post hoc analysis from the SURPASS Clinical Trial Program

**DOI:** 10.1186/s12933-024-02147-9

**Published:** 2024-02-10

**Authors:** Stephen J. Nicholls, Santiago Tofé, Carel W. le Roux, David A. D’Alessio, Russell J. Wiese, Imre Pavo, Katelyn Brown, Govinda J. Weerakkody, Meltem Zeytinoglu, Irene C. Romera

**Affiliations:** 1 Victorian Heart Institute, Monash University, Clayton, VIC Australia; 2 Department of Endocrinology and Nutrition, University Hospital Son Espases, Palma, Spain; 3 Diabetes Complications Research Centre, Conway Institute, School of Medicine, University College Dublin, Dublin, Ireland; 4 Diabetes Research Centre, Ulster University, Coleraine, UK; 5 Division of Endocrinology, Department of Medicine, Duke Molecular Physiology Institute, Duke University, Durham, NC USA; 6 Eli Lilly and Company, Indianapolis, IN USA; 7 Eli Lilly Regional Operations GmbH, Vienna, Austria; 8 Eli Lilly and Company, Avda. de La Industria 30, 28108 Alcobendas, Madrid, Spain

**Keywords:** Metabolic syndrome, Type 2 diabetes, Tirzepatide, Incretin

## Abstract

**Background:**

Metabolic syndrome is characterized as the co-occurrence of interrelated cardiovascular risk factors, including insulin resistance, hyperinsulinemia, abdominal obesity, dyslipidemia and hypertension. Once weekly tirzepatide is approved in the US and EU for the treatment of type 2 diabetes (T2D) and obesity. In the SURPASS clinical trial program for T2D, tirzepatide demonstrated greater improvements in glycemic control, body weight reduction and other cardiometabolic risk factors versus placebo, subcutaneous semaglutide 1 mg, insulin degludec, and insulin glargine. This post hoc analysis assessed the effect of tirzepatide use on the prevalence of patients meeting the criteria for metabolic syndrome across SURPASS 1–5.

**Methods:**

Metabolic syndrome was defined as having ≥ 3 of 5 criteria according to the US National Cholesterol Education Program: Adult Treatment Panel III. Analyses were based on on-treatment data at the primary endpoint from patients adherent to treatment (taking ≥ 75% study drug). A logistic regression model with metabolic syndrome status as the response variable, metabolic syndrome status at the baseline visit as an adjustment, and randomized treatment as fixed explanatory effect was used. The effect of tirzepatide use on the prevalence of patients meeting the criteria for metabolic syndrome by categorical weight loss, background medication and gender were assessed.

**Results:**

In SURPASS, the prevalence of patients meeting the criteria for metabolic syndrome at baseline was 67–88% across treatment groups with reductions at the primary endpoint to 38–64% with tirzepatide versus 64–82% with comparators. Reductions in the prevalence of patients meeting the criteria for metabolic syndrome was significantly greater with all tirzepatide doses versus placebo, semaglutide 1 mg, insulin glargine, and insulin degludec (p < 0.001). Individual components of metabolic syndrome were also reduced to a greater extent with tirzepatide vs comparators. Greater reductions in body weight were associated with greater reductions in the prevalence of patients meeting the criteria for metabolic syndrome and its individual components. Background SGLT2i or sulfonylurea use or gender did not impact the change in prevalence of patients meeting the criteria for metabolic syndrome.

**Conclusions:**

In this post hoc analysis, tirzepatide at all doses studied was associated with a greater reduction in the prevalence of patients meeting the criteria for metabolic syndrome compared to placebo, semaglutide 1 mg, insulin degludec, and insulin glargine. Although more evidence is needed, these data would support greater potential improvement in cardiovascular risk factor profile with tirzepatide treatment in people across the continuum of T2D.

**Supplementary Information:**

The online version contains supplementary material available at 10.1186/s12933-024-02147-9.

## Introduction

In his 1988 Banting lecture, Reaven [1] referred to “Syndrome X” as the presence of insulin resistance, hyperglycemia, hyperinsulinemia, increased plasma concentration of very low-density lipoprotein (VLDL) triglyceride, decreased plasma concentration of high-density lipoprotein (HDL) cholesterol, and high blood pressure occurring in the same individual. He further hypothesized that “Syndrome X”, or what is referred to today as metabolic syndrome, may be linked to the development of coronary artery disease (CAD).

In recent years, the prevalence of obesity has increased [2, 3], resulting in a greater prevalence of people meeting the criteria for metabolic syndrome. Obesity and metabolic syndrome are associated with increased risk of CAD and other major adverse cardiovascular events (MACE) [4–9]. Reduction in body weight can improve metabolic syndrome and its individual components [10–15]. Therefore, lifestyle modifications are recommended as the first line of treatment for metabolic syndrome [16]. However, lifestyle modifications alone are often not enough to achieve and maintain significant body weight reduction in the long term. Furthermore, in the Look AHEAD (Action for Health in Diabetes), the effect of an intensive lifestyle intervention was assessed in people with a body mass index (BMI) ≥ 25 kg/m^2^ and type 2 diabetes (T2D) [17]. Although patients maintained a 6% weight loss during a 10-year period, weight loss did not reduce the rate of cardiovascular events. In the sub population of patients who lost more than 10% body weight, a reduction in cardiovascular events was seen [18].

Bariatric surgery also improves glycemic control and reduces cardiovascular risk factors. In the STAMPEDE trial, in people with T2D and a body mass index (BMI) of 27–43 kg/m^2^, both gastric bypass and sleeve gastrectomy in combination with intensive medical therapy were superior to intensive medical therapy alone in achieving glycated hemoglobin (HbA1c) ≤ 6.0%, [19–21], reducing cardiovascular risk and medication use [22], and thus, improving quality of life [20, 21]. Furthermore, approximately 92% of patients overall had metabolic syndrome at baseline, but 1 year after the interventions, the surgical groups had better metabolic improvement compared to the intensive medical therapy group. At 1 year, resolution of metabolic syndrome occurred in 35% of patients in the intensive medical therapy group, 65% in the gastric bypass group, and 59% in the sleeve gastrectomy group [19].

Tirzepatide is a once-weekly subcutaneous injection of glucose-dependent insulinotropic polypeptide (GIP) and glucagon-like peptide-1 (GLP-1) receptor agonist approved in the US and EU for the treatment of T2D and obesity. In the SURPASS clinical trial program, treatment with tirzepatide at all doses (5 mg, 10 mg, and 15 mg) resulted in substantial reductions in HbA1c, ranging from − 1.9% to − 2.6%, and robust body weight reductions, ranging from − 6.6% to − 13.9% over a treatment period of 40 to 104 weeks [23–27]. Moreover, this finding was extended in a 72-week trial in participants with obesity and T2D, where tirzepatide 10 and 15 mg resulted in body weight reductions of 13.4% and 15.7%, respectively, versus 3.3% with placebo. [28]. The safety profile of tirzepatide is consistent with that of GLP-1 receptor agonists, with the most commonly reported adverse events being generally mild to moderate gastrointestinal symptoms, which typically decreased over time. Tirzepatide treatment has also significantly improved markers of beta-cell function and insulin sensitivity as monotherapy and compared to dulaglutide 1.5 mg and semaglutide 1 mg [29–31] and demonstrated cardiovascular safety when compared with pooled comparators for MACE-4, including death due to cardiovascular cause, myocardial infarction, stroke and hospitalization for unstable angina [32]. In the phase 2b trial, tirzepatide at the 15 mg dose administered over 26 weeks uniquely modulated 54 metabolites associated with T2D risk and metabolic dysregulation consistent with improved cardiovascular risk factor profile, compared to only 6 metabolites with dulaglutide, and no changes with placebo [33].

This post hoc analysis assessed the effect of tirzepatide use on the prevalence of patients meeting the criteria for metabolic syndrome across the five SURPASS registrational clinical trials and the association between tirzepatide-induced weight loss and the prevalence of patients meeting the criteria for metabolic syndrome.

## Materials and methods

### Trial design and study population

The trial designs, study populations and primary results of SURPASS-1, SURPASS-2, SURPASS-3, SURPASS-4 and SURPASS-5 are published [23–27]. Two of the trials were placebo-controlled (SURPASS-1 and SURPASS-5) while the remaining three trials compared tirzepatide to semaglutide 1 mg (SURPASS-2), titrated insulin degludec (SURPASS-3) and titrated insulin glargine (SURPASS-4). The Phase 3 SURPASS clinical trial program included 6278 patients and treatment periods ranging from 40 to 104 weeks. The SURPASS registrational clinical trials were designed to evaluate the safety and efficacy of tirzepatide (5 mg, 10 mg, or 15 mg) in adults aged 18 years or older, with T2D (baseline HbA1c ≥ 7.0 or ≥ 7.5% to ≤ 9.5% or ≤ 10.5% [53–91 mmol/mol] and BMI ≥ 23 kg/m^2^ or ≥ 25 kg/m^2^, depending on individual trial criteria). The primary efficacy measure was HbA1c reduction from baseline at the primary endpoints of 40 or 52 weeks, depending on the individual trial, with an objective of superiority of tirzepatide versus placebo or non-inferiority of tirzepatide compared with active comparators. There were no diet and exercise recommendations beyond the usual practice at each study center and concomitant pharmacotherapy that promoted weight loss was not allowed. All laboratory parameters were assessed in a central laboratory.

The trials assessed in this analysis (NCT03954834, NCT03987919, NCT03882970, NCT03730662, and NCT04039503) were conducted in accordance with the International Conference on Harmonisation Guidelines for Good Clinical Practice and the Declaration of Helsinki. All patients provided signed informed consent and protocols were approved by local ethical review boards.

### Categorical cut-off points for meeting the criteria for metabolic syndrome

A list of the criteria used to define metabolic syndrome in this report is presented in Additional file 1: Table S1. The definition of metabolic syndrome was based on the NCEP ATP III (2005 revision) criteria (AHA/NHLBI) [34, 35]. The individual component of hyperglycemia was defined as either fasting serum glucose ≥ 100 mg/dL (5.6 mmol/L) (NCEP ATP III) or HbA1c ≥ 5.7% (38.8 mmol/mol) to increase accuracy [16].

### Outcomes

The prevalence of patients meeting the criteria for metabolic syndrome and individual components of metabolic syndrome in the SURPASS clinical trial program was assessed at baseline and at the primary endpoint of Week 40 (SURPASS-1, SURPASS-2 and SURPASS-5) or 52 Week (SURPASS-3 and SURPASS-4) in patients treated with tirzepatide, placebo, or active comparators. Subgroup analyses of the effect of tirzepatide use on the prevalence of patients meeting the criteria for metabolic syndrome by categorical weight loss (< 5%, > 5% to ≤ 10%, > 10% to ≤ 15%, > 15% to ≤ 20%, or > 20%, and < 15% or ≥ 15%) and background medication use (sodium-glucose co-transporter 2 inhibitors [SGLT2i] or sulfonylurea) and gender were also assessed. Subgroup analyses were not performed on patients on background metformin. All laboratory parameters were assessed in a central laboratory.

### Statistical analysis

Analyses was conducted, separately using baseline data and on treatment data at the study primary endpoint visit for five randomized controlled trials using a cohort of patient deemed compliant to study drug (taking ≥ 75% of assigned study drug). A logistic regression model with metabolic syndrome status (yes, no) at primary endpoint visit of the study as the response variable with metabolic syndrome status (yes, no) at the baseline visit as an adjustment and randomized treatment as fixed explanatory effect was used to compare prevalence of patients meeting the criteria for metabolic syndrome at the primary endpoint visit between tirzepatide and comparator. All analyses presented are exploratory in nature, and a p-value < 0.05 was considered statistically significant. Analyses were performed using SAS version 9.4 (Copyright © 2017 SAS Institute Inc., Cary, NC, USA).

## Results

### Patient disposition and characteristics at baseline

A total of 5219 patients were included in this post hoc analysis (tirzepatide 5 mg, N = 1206, tirzepatide 10 mg, N = 1162, tirzepatide 15 mg, N = 1046, comparator, N = 1805). Clinical characteristics and baseline demographics were well balanced between tirzepatide and comparators for each study [22–26]. At baseline, 4056 patients (78%) met the criteria of having metabolic syndrome (tirzepatide 5 mg, n = 974; tirzepatide 10 mg, n = 979; tirzepatide 15 mg, n = 960; comparator, n = 1476). Similar prevalence of patients meeting the criteria for metabolic syndrome was observed despite different stages of T2D across the SURPASS clinical trial program (Table 1).

**Table 1:** Prevalence of patients meeting the criteria for metabolic syndrome in the SURPASS clinical trial program

Metabolic Syndrome Risk Factors	Tirzepatide 5 mg		Tirzepatide 10 mg		Tirzepatide 15 mg		Comparator	
	Baseline	Primary Endpoint	Baseline	Primary Endpoint	Baseline	Primary Endpoint	Baseline	Primary Endpoint
*SURPASS-1 monotherapy, N*	**105**		**103**		**93**		**95**	
≥ 3 Risk Factors	77 (73.3)	58 (55.2)	69 (67.0)	49 (47.6)	70 (75.3)	36 (38.7)	73 (76.8)	68 (71.6)
WC > 102 cm (M), > 89 cm (F)	76 (72.4)	63 (60.0)	67 (65.0)	47 (45.6)	61 (65.6)	46 (49.5)	64 (67.4)	61 (64.2)
FSG ≥ 100 mg/dL or HbA1c ≥ 5.7%	105 (100.0)	83 (79.0)	103 (100.0)	84 (81.6)	93 (100.0)	60 (64.5)	95 (100.0)	94 (98.9)
SBP > 130 mmHg or DBP > 85 mmHg	50 (47.6)	40 (38.1)	45 (43.7)	29 (28.2)	44 (47.3)	26 (28.0)	41 (43.2)	41 (43.2)
Triglycerides > 150 mg/dL	52 (49.5)	37 (35.2)	52 (50.5)	31 (30.1)	45 (48.4)	23 (24.7)	53 (55.8)	48 (50.5)
HDL < 40 mg/dL (M), < 50 mg/dL (F)	62 (59.0)	53 (50.5)	51 (49.5)	50 (48.5)	55 (59.1)	47 (50.5)	55 (57.9)	56 (58.9)
*SURPASS-2 add-on to MET vs SEMA 1 mg, N*	**415**		**387**		**389**		**414**	
≥ 3 Risk Factors	341 (82.2)	227 (54.7)	336 (86.8)	199 (51.4)	328 (84.3)	161 (41.4)	344 (83.1)	266 (64.3)
WC > 102 cm (M), > 89 cm (F)	345 (83.1)	272 (65.5)	326 (84.2)	244 (63.0)	326 (83.8)	234 (60.2)	349 (84.3)	278 (67.1)
FSG ≥ 100 mg/dL or HbA1c ≥ 5.7%	415 (100.0)	345 (83.1)	387 (100.0)	263 (68.0)	389 (100.0)	235 (60.4)	414 (100.0)	358 (86.5)
SBP > 130 mmHg or DBP > 85 mmHg	230 (55.4)	165 (39.8)	244 (63.0)	148 (38.2)	206 (53.0)	139 (35.7)	234 (56.5)	187 (45.2)
Triglycerides > 150 mg/dL	227 (54.7)	159 (38.3)	211 (54.5)	132 (34.1)	209 (53.7)	120 (30.8)	225 (54.3)	193 (46.6)
HDL < 40 mg/dL (M), < 50 mg/dL (F)	230 (55.4)	183 (44.1)	216 (55.8)	170 (43.9)	229 (58.9)	176 (45.2)	239 (57.7)	201 (48.6)
SURPASS-3 add-on to *MET ± SGLT2i vs iDeg, N*	**308**		**291**		**292**		**313**	
≥ 3 Risk Factors	245 (79.5)	184 (59.7)	249 (85.6)	139 (47.8)	249 (85.3)	145 (49.7)	242 (77.3)	248 (79.2)
WC > 102 cm (M), > 89 cm (F)	255 (82.8)	212 (68.8)	248 (85.2)	170 (58.4)	252 (86.3)	173 (59.2)	253 (80.8)	263 (84.0)
FSG ≥ 100 mg/dL or HbA1c ≥ 5.7%	308 (100.0)	269 (87.3)	291 (100.0)	220 (75.6)	292 (100.0)	211 (72.3)	313 (100.0)	297 (94.9)
SBP > 130 mmHg or DBP > 85 mmHg	185 (60.1)	131 (42.5)	173 (59.5)	109 (37.5)	180 (61.6)	112 (38.4)	201 (64.2)	192 (61.3)
Triglycerides > 150 mg/dL	166 (53.9)	120 (39.0)	155 (53.3)	91 (31.3)	163 (55.8)	101 (34.6)	155 (49.5)	129 (41.2)
HDL < 40 mg/dL (M), < 50 mg/dL (F)	153 (49.7)	135 (43.8)	171 (58.8)	118 (40.5)	163 (55.8)	125 (42.8)	154 (49.2)	150 (47.9)
*SURPASS-4 ± MET ± SGLT2i ± SU vs iGlar, N*	**273**		**276**		**274**		**870**	
≥ 3 Risk Factors	227 (83.2)	159 (58.2)	242 (87.7)	163 (59.1)	237 (86.5)	141 (51.5)	727 (83.6)	717 (82.4)
WC > 102 cm (M), > 89 cm (F)	222 (81.3)	174 (63.7)	227 (82.2)	163 (59.1)	226 (82.5)	158 (57.7)	674 (77.5)	709 (81.5)
FSG ≥ 100 mg/dL or HbA1c ≥ 5.7%	273 (100.0)	227 (83.2)	276 (100.0)	227 (82.2)	274 (100.0)	193 (70.4)	869 (99.9)	843 (96.9)
SBP > 130 mmHg or DBP > 85 mmHg	169 (61.9)	134 (49.1)	180 (65.2)	155 (56.2)	179 (65.3)	138 (50.4)	590 (64.4)	609 (70.0)
Triglycerides > 150 mg/dL	154 (56.4)	112 (41.0)	146 (52.9)	92 (33.3)	151 (55.1)	92 (33.6)	451 (51.8)	416 (47.8)
HDL < 40 mg/dL (M), < 50 mg/dL (F)	156 (57.1)	130 (47.6)	182 (65.9)	130 (47.1)	168 (61.3)	124 (45.3)	509 (58.5)	466 (53.6)
*SURPASS-5 add-on to insulin glargine ± MET vs PBO, N*	**105**		**105**		**98**		**113**	
≥ 3 Risk Factors	84 (80.0)	67 (63.8)	83 (79.0)	55 (52.4)	76 (77.6)	37 (37.8)	90 (79.6)	90 (79.6)
WC > 102 cm (M), > 89 cm (F)	92 (87.6)	79 (75.2)	87 (82.9)	76 (72.4)	81 (82.7)	69 (70.4)	90 (79.6)	92 (81.4)
FSG ≥ 100 mg/dL or HbA1c ≥ 5.7%	105 (100.0)	84 (80.0)	105 (100.0)	68 (64.8)	98 (100.0)	54 (55.1)	113 (100.0)	110 (97.3)
SBP > 130 mmHg or DBP > 85 mmHg	73 (69.5)	59 (56.2)	78 (74.3)	55 (52.4)	69 (70.4)	31 (31.6)	84 (74.3)	80 (70.8)
Triglycerides > 150 mg/dL	48 (45.7)	33 (31.4)	52 (49.5)	31 (29.5)	37 (37.8)	17 (17.3)	48 (42.5)	44 (38.9)
HDL < 40 mg/dL (M), < 50 mg/dL (F)	53 (50.5)	47 (44.8)	45 (42.9)	39 (37.1)	43 (43.9)	40 (40.8)	46 (40.7)	46 (40.7)

Data are n (%) at baseline and at the primary endpoint of 40 weeks (SURPASS-1, SURPASS-2 and SURPASS-5) or 52 weeks (SURPASS-3, SURPASS-4) in patients on-treatment compliant to study drug (patients taking ≥ 75% of assigned doses)

DBP: diastolic blood pressure; F: female; FSG: fasting serum glucose; HbA1c: glycated hemoglobin; HDL: high-density lipoprotein; iDeg: insulin degludec; iGlar: insulin glargine; M: male; MET: metformin; n: number of patients in the specified category; PBO: placebo; SBP: systolic blood pressure; SEMA: semaglutide; SGLT2i: sodium glucose cotransporter 2 inhibitor; SU: sulfonylurea; WC: waist circumference

### Effect of tirzepatide use on the prevalence of metabolic syndrome in the SURPASS clinical trial program

**Fig. 1:**
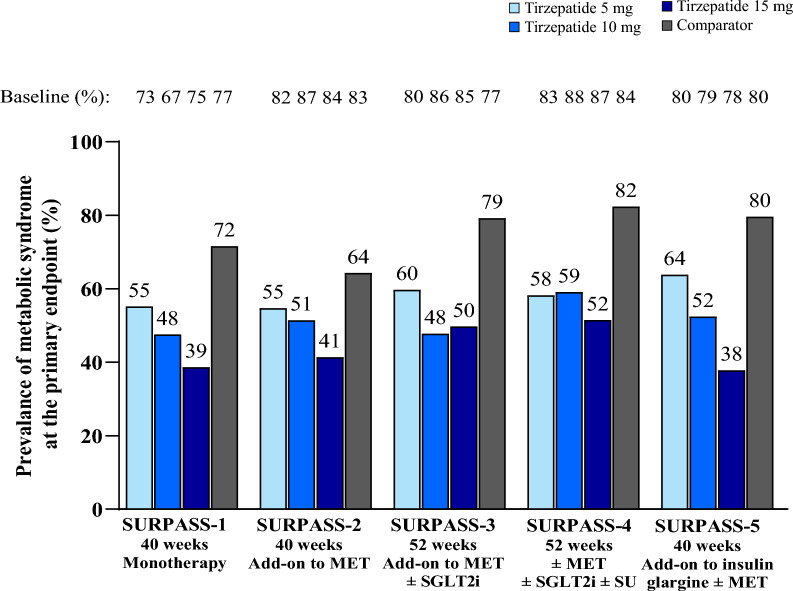
Prevalence of patients meeting the criteria for metabolic syndrome in the SURPASS clinical trial program. Data are proportion of patients with at least 3 diagnostic criteria for metabolic syndrome at the primary endpoint of 40/52 weeks. Pooled tirzepatide vs comparator was statistically significant in each trial. MET: metformin; SGLT2i: sodium-glucose co-transporter 2 inhibitor; SU: sulfonylurea

Overall, the prevalence of patients meeting the criteria for metabolic syndrome was reduced with tirzepatide across the SURPASS clinical trial program and was dose-dependent, with the greatest reductions observed with tirzepatide 15 mg. The proportion of patients with at least 3 criteria for metabolic syndrome ranged from 67–88% at baseline to 38–64% at Week 40/52 with tirzepatide versus 77–84% to 64–82% with comparators, respectively (Table 1 and Fig. 1). The reduction in the prevalence of patients meeting the criteria for metabolic syndrome was significantly greater with pooled tirzepatide doses compared to placebo, semaglutide 1 mg, insulin glargine, and insulin degludec (p < 0.001, all comparisons). With the exception of the semaglutide 1 mg arm (from 83% at baseline to 64% at the primary endpoint), the prevalence of patients meeting the criteria for metabolic syndrome in the comparator groups remained unchanged from baseline to primary endpoint visit.

### Effect of tirzepatide use on individual components of metabolic syndrome

Treatment with tirzepatide improved individual components of metabolic syndrome (Table 1). The prevalence of waist circumference > 102 cm in males and > 89 cm in females reduced from 65–88% at baseline to 46–75% at Week 40/52 with tirzepatide versus 67–84% to 64–84% with comparators, respectively. The prevalence of fasting serum glucose ≥ 5.6 mmol/L (100 mg/dL) or HbA1c ≥ 38.8 mmol/mol (5.7%) reduced from 100% at baseline to 55–87% with tirzepatide versus 87–99% with comparators at Week 40/52. The prevalence of systolic blood pressure ≥ 130 mmHg or diastolic blood pressure ≥ 85 mmHg reduced from 44–74% at baseline to 28–56% at Week 40/52 with tirzepatide versus 43–74% to 43–71% with comparators, respectively. The prevalence of triglycerides > 150 mg/dL reduced from 38–56% at baseline to 17–41% at Week 40/52 with tirzepatide versus 43–56% to 39–51% with comparators, respectively. Modest reductions in the prevalence of HDL < 40 mg/dL in males and < 50 mg/dL in females were observed with tirzepatide, ranging from 43–66% at baseline to 37–51% at Week 40/52 vs 41–59% to 41–59% with comparators, respectively.

### Association of tirzepatide use on the prevalence of patients meeting the criteria for metabolic syndrome by weight loss category, SGLT2i or sulfonylurea use, or gender

**Fig. 2:**
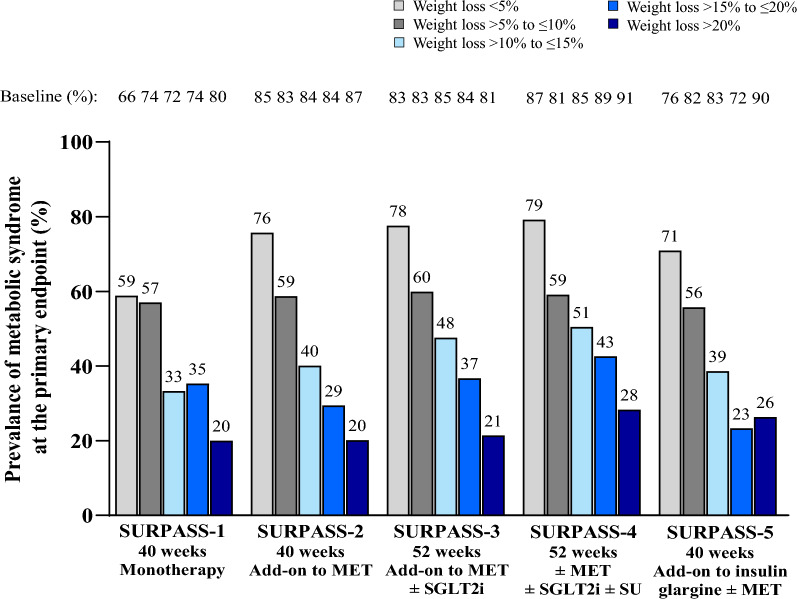
Prevalence of patients meeting the criteria for metabolic syndrome in tirzepatide-treated patients by weight loss category (< 5%, > 5% to ≤ 10%, > 10% to ≤ 15%, > 15% to ≤ 20%, > 20%). Data are proportion of tirzepatide-treated patients with at least 3 diagnostic criteria for metabolic syndrome at the primary endpoint of 40/52 weeks by weight loss category (< 5%, > 5% to ≤ 10%, > 10% to ≤ 15%, > 15% to ≤ 20%, > 20%). MET: metformin; SGLT2i: sodium-glucose co-transporter 2 inhibitor; SU: sulfonylurea

**Fig. 3:**
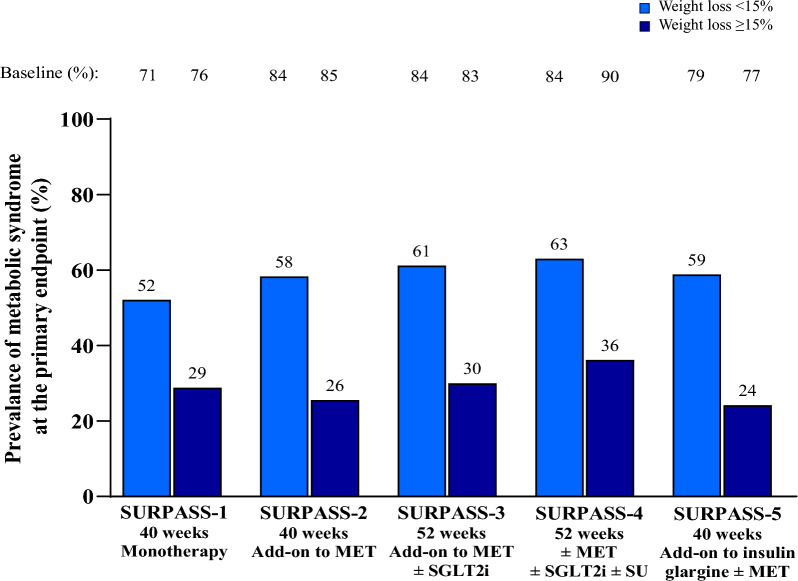
Prevalence of patients meeting the criteria for metabolic syndrome in tirzepatide-treated patients by weight loss category (< 15% or ≥ 15%). Data are proportion of tirzepatide-treated patients with at least 3 diagnostic criteria for metabolic syndrome at the primary endpoint of 40/52 weeks by weight loss category (< 15% or ≥ 15%). MET: metformin; SGLT2i: sodium-glucose co-transporter 2 inhibitor; SU: sulfonylurea

**Fig. 4:**
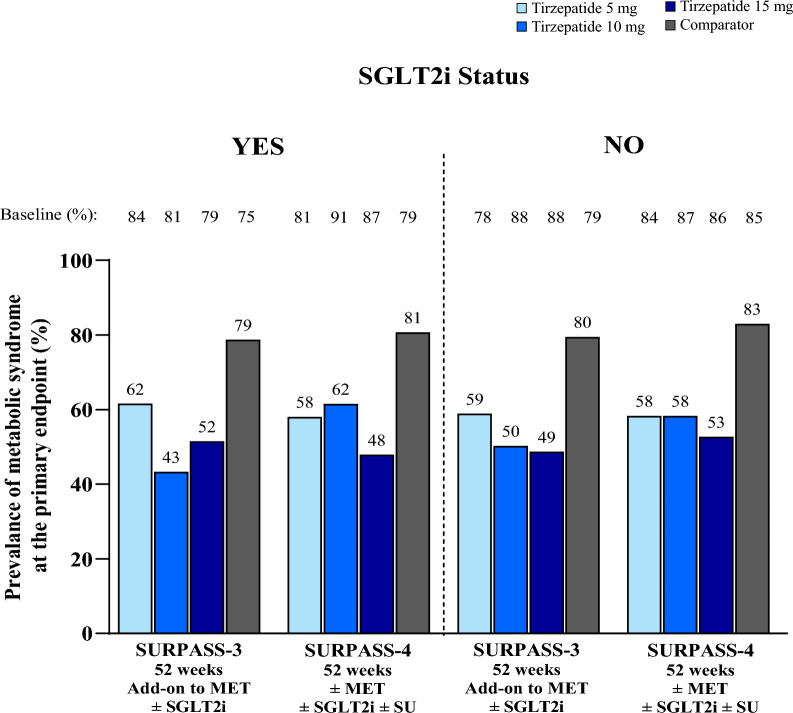
Prevalence of patients meeting the criteria for metabolic syndrome by SGLT2i status (yes, no). Data are proportion of tirzepatide-treated patients with at least 3 diagnostic criteria for metabolic syndrome at the primary endpoint of 40/52 weeks by SGLT2i status (yes, no). MET: metformin; SGLT2i: sodium-glucose co-transporter 2 inhibitor; SU: sulfonylurea

In the SURPASS clinical trial program, greater body weight reduction with tirzepatide was associated with greater reduction in the prevalence of patients meeting the criteria for metabolic syndrome. In patients who had > 20% body weight reduction, the prevalence of patients meeting the criteria for metabolic syndrome decreased from 80–91% at baseline to 20–28% at Week 40/52 (Table 2 and Fig. 2). Furthermore, patients achieving ≥ 15% body weight reduction demonstrated a greater reduction in the prevalence of the majority of individual components, albeit to a lesser extent for changes in HDL cholesterol (Fig. 3 and Additional file 1: Table S2). These finding were unaffected by background use of SGLT2i (Fig. 4 and Additional file 1: Table S3), background use of sulfonylurea (Table 3) and was not meaningfully different between females and males (Additional file 1: Figures S1 and S2).

**Table 2:** Prevalence of patients meeting the criteria for metabolic syndrome in tirzepatide-treated patients by weight loss category (< 5%, > 5% to ≤ 10%, > 10% to ≤ 15%, > 15% to ≤ 20%, or > 20%)

Metabolic syndrome risk factors	Weight Loss < 5%		Weight Loss > 5% to ≤ 10%		Weight Loss > 10% to ≤ 15%		Weight Loss > 15% to ≤ 20%		Weight Loss > 20%	
	Baseline	Primary Endpoint	Baseline	Primary Endpoint	Baseline	Primary Endpoint	Baseline	Primary Endpoint	Baseline	Primary Endpoint
*SURPASS-1 monotherapy, N*	**85**		**100**		**57**		**34**		**25**	
≥ 3 Risk Factors	56 (65.9)	50 (58.8)	74 (74.0)	57 (57.0)	41 (71.9)	19 (33.3)	25 (73.5)	12 (35.3)	20 (80.0)	5 (20.0)
WC > 102 cm (M), > 89 cm (F)	57 (67.1)	53 (62.4)	69 (69.0)	52 (52.0)	35 (61.4)	24 (42.1)	24 (70.6)	16 (47.1)	19 (76.0)	11 (44.0)
FSG ≥ 100 mg/dL or HbA1c ≥ 5.7%	85 (100.0)	73 (85.9)	100 (100.0)	86 (86.0)	57 (100.0)	38 (66.7)	34 (100.0)	20 (58.8)	25 (100.0)	10 (40.0)
SBP > 130 mmHg or DBP > 85 mmHg	47 (55.3)	36 (42.4)	38 (38.0)	34 (34.0)	27 (47.4)	14 (24.6)	19 (55.9)	9 (26.5)	8 (32.0)	2 (8.0)
Triglycerides > 150 mg/dL	38 (44.7)	28 (32.9)	55 (55.0)	41 (41.0)	30 (52.6)	15 (26.3)	14 (41.2)	5 (14.7)	12 (48.0)	2 (8.0)
HDL < 40 mg/dL (M), < 50 mg/dL (F)	40 (47.1)	40 (47.1)	58 (58.0)	51 (51.0)	33 (57.9)	27 (47.4)	20 (58.8)	19 (55.9)	17 (68.0)	13 (52.0)
*SURPASS-2 add-on to MET vs SEMA 1 mg, N*	**268**		**327**		**269**		**187**		**139**	
≥ 3 Risk Factors	227 (84.7)	203 (75.7)	272 (83.2)	192 (58.7)	227 (84.4)	108 (40.1)	157 (84.0)	55 (29.4)	121 (87.1)	28 (20.1)
WC > 102 cm (M), > 89 cm (F)	222 (82.8)	208 (77.6)	268 (82.0)	224 (68.5)	224 (83.3)	162 (60.2)	158 (84.5)	96 (51.3)	124 (89.2)	59 (42.4)
FSG ≥ 100 mg/dL or HbA1c ≥ 5.7%	268 (100.0)	254 (94.8)	327 (100.0)	273 (83.5)	269 (100.0)	180 (66.9)	187 (100.0)	89 (47.6)	139 (100.0)	46 (33.1)
SBP > 130 mmHg or DBP > 85 mmHg	160 (59.7)	145 (54.1)	191 (58.4)	139 (42.5)	149 (55.4)	85 (31.6)	100 (53.5)	50 (26.7)	79 (56.8)	32 (23.0)
Triglycerides > 150 mg/dL	145 (54.1)	130 (48.5)	189 (57.8)	130 (39.8)	151 (56.1)	83 (30.9)	98 (52.4)	41 (21.9)	64 (46.0)	27 (19.4)
HDL < 40 mg/dL (M), < 50 mg/dL (F)	148 (55.2)	126 (47.0)	177 (54.1)	151 (46.2)	159 (59.1)	109 (40.5)	110 (58.8)	89 (47.6)	80 (57.6)	53 (38.1)
*SURPASS-3 add-on to MET ± SGLT2i vs iDeg, N*	**210**		**207**		**229**		**128**		**117**	
≥ 3 Risk Factors	174 (82.9)	163 (77.6)	171 (82.6)	124 (59.9)	195 (85.2)	109 (47.6)	108 (84.4)	47 (36.7)	95 (81.2)	25 (21.4)
WC > 102 cm (M), > 89 cm (F)	181 (86.2)	171 (81.4)	171 (82.6)	134 (64.7)	187 (81.7)	135 (59.0)	108 (84.4)	66 (51.6)	108 (92.3)	49 (41.9)
FSG ≥ 100 mg/dL or HbA1c ≥ 5.7%	210 (100.0)	205 (97.6)	207 (100.0)	181 (87.4)	229 (100.0)	182 (79.5)	128 (100.0)	82 (64.1)	117 (100.0)	50 (42.7)
SBP > 130 mmHg or DBP > 85 mmHg	121 (57.6)	107 (51.0)	125 (60.4)	97 (46.9)	155 (67.7)	81 (35.4)	71 (55.5)	39 (30.5)	66 (56.4)	28 (23.9)
Triglycerides > 150 mg/dL	120 (57.1)	120 (57.1)	109 (52.7)	81 (39.1)	121 (52.8)	65 (28.4)	75 (58.6)	32 (25.0)	59 (50.4)	14 (12.0)
HDL < 40 mg/dL (M), < 50 mg/dL (F)	106 (50.5)	103 (49.0)	117 (56.5)	90 (43.5)	121 (52.8)	90 (39.3)	79 (61.7)	56 (43.8)	64 (54.7)	39 (33.3)
*SURPASS-4 ± MET ± SGLT2i ± SU vs iGlar, N*	**212**		**186**		**218**		**115**		**92**	
≥ 3 Risk Factors	184 (86.8)	168 (79.2)	151 (81.2)	110 (59.1)	185 (84.9)	110 (50.5)	102 (88.7)	49 (42.6)	84 (91.3)	26 (28.3)
WC > 102 cm (M), > 89 cm (F)	177 (83.5)	170 (80.2)	142 (76.3)	107 (57.5)	175 (80.3)	117 (53.7)	95 (82.6)	64 (55.7)	86 (93.5)	37 (40.2)
FSG ≥ 100 mg/dL or HbA1c ≥ 5.7%	212 (100.0)	206 (97.2)	186 (100.0)	167 (89.8)	218 (100.0)	165 (75.7)	115 (100.0)	68 (59.1)	92 (100.0)	41 (44.6)
SBP > 130 mmHg or DBP > 85 mmHg	141 (66.5)	130 (61.3)	113 (60.8)	84 (45.2	149 (68.3)	110 (50.5)	73 (63.5)	67 (58.3)	52 (56.5)	36 (39.1)
Triglycerides > 150 mg/dL	122 (57.5)	96 (45.3)	113 (60.8)	77 (41.4)	111 (50.9)	70 (32.1)	64 (55.7)	36 (31.3)	41 (44.6)	17 (18.5)
HDL < 40 mg/dL (M), < 50 mg/dL (F)	124 (58.5)	119 (56.1)	110 (59.1)	87 (46.8)	131 (60.1)	96 (44.0)	78 (67.8)	47 (40.9)	63 (68.5)	35 (38.0)
*SURPASS-5 add-on to insulin glargine ± MET vs PBO, N*	**110**		**79**		**57**		**43**		**19**	
≥ 3 Risk Factors	83 (75.5)	78 (70.9)	65 (82.3)	44 (55.7)	47 (82.5)	22 (38.6)	31 (72.1)	10 (23.3)	17 (89.5)	5 (26.3)
WC > 102 cm (M), > 89 cm (F)	96 (87.3)	93 (84.5)	67 (84.8)	60 (75.9)	48 (84.2)	41 (71.9)	32 (74.4)	19 (44.2)	17 (89.5)	11 (57.9)
FSG ≥ 100 mg/dL or HbA1c ≥ 5.7%	110 (100.0)	98 (89.1)	79 (100.0)	56 (70.9)	57 (100.0)	35 (61.4)	43 (100.0)	15 (34.9)	19 (100.0)	2 (10.5)
SBP > 130 mmHg or DBP > 85 mmHg	77 (70.0)	72 (65.5)	55 (69.6)	40 (50.6)	43 (75.4)	15 (26.3)	31 (72.1)	13 (30.2)	14 (73.7)	5 (26.3)
Triglycerides > 150 mg/dL	44 (40.0)	34 (30.9)	40 (50.6)	24 (30.4)	24 (42.1)	13 (22.8)	17 (39.5)	6 (14.0)	12 (63.2)	4 (21.1)
HDL < 40 mg/dL (M), < 50 mg/dL (F)	49 (44.5)	48 (43.6)	43 (54.4)	37 (46.8)	25 (43.9)	20 (35.1)	14 (32.6)	14 (32.6)	10 (52.6)	7 (36.8)

Data are n (%) at baseline and at the primary endpoint of 40 weeks (SURPASS-1, SURPASS-2 and SURPASS-5) or 52 weeks (SURPASS-3, SURPASS-4) in patients on-treatment compliant to study drug (patients taking ≥ 75% of assigned doses)

DBP: diastolic blood pressure; F: female; FSG: fasting serum glucose; HbA1c: glycated hemoglobin; HDL: high-density lipoprotein; iDeg: insulin degludec; iGlar: insulin glargine; M: male; MET: metformin; n: number of patients in the specified category; PBO: placebo; SBP: systolic blood pressure; SEMA: semaglutide; SGLT2i: sodium glucose cotransporter 2 inhibitor; SU: sulfonylurea; WC: waist circumference

**Table 3:** Prevalence of patients meeting the criteria for metabolic syndrome by sulfonylurea status (yes, no)

Metabolic Syndrome Risk Factors	Tirzepatide 5 mg		Tirzepatide 10 mg		Tirzepatide 15 mg		Comparator	
	Baseline	Primary Endpoint	Baseline	Primary Endpoint	Baseline	Primary Endpoint	Baseline	Primary Endpoint
*SURPASS-4 ± MET ± SGLT2i ± SU vs iGlar, N, yes*	**156**		**157**		**155**		**466**	
≥ 3 Risk Factors	128 (82.1)	91 (58.3)	141 (89.8)	96 (61.1)	140 (90.3)	84 (54.2)	396 (85.0)	392 (84.1)
WC > 102 cm (M), > 89 cm (F)	124 (79.5)	100 (64.1)	129 (82.2)	96 (61.1)	128 (82.6)	91 (58.7)	359 (77.0)	385 (82.6)
FSG ≥ 100 mg/dL or HbA1c ≥ 5.7%	156 (100.0)	129 (82.7)	157 (100.0)	133 (84.7)	155 (100.0)	119 (76.8)	466 (100.0)	460 (98.7)
SBP > 130 mmHg or DBP > 85 mmHg	96 (61.5)	76 (48.7)	107 (68.2)	85 (54.1)	107 (69.0)	81 (52.3)	296 (63.5)	333 (71.5)
Triglycerides > 150 mg/dL	93 (59.6)	68 (43.6)	82 (52.2)	54 (34.4)	95 (61.3)	51 (32.9)	248 (53.2)	230 (49.4)
HDL < 40 mg/dL (M), < 50 mg/dL (F)	92 (59.0)	77 (49.4)	102 (65.0)	77 (49.0)	94 (60.6)	70 (45.2)	279 (59.9)	261 (56.0)
*SURPASS-4 ± MET ± SGLT2i ± SU vs iGlar, N, no*	**117**		**119**		**119**		**404**	
≥ 3 Risk Factors	99 (84.6)	68 (58.1)	101 (84.9)	67 (56.3)	97 (81.5)	57 (47.9)	331 (81.9)	325 (80.4)
WC > 102 cm (M), > 89 cm (F)	98 (83.8)	74 (63.2)	98 (82.4)	67 (56.3)	98 (82.4)	67 (56.3)	315 (78.0)	324 (80.2)
FSG ≥ 100 mg/dL or HbA1c ≥ 5.7%	117 (100.0)	98 (83.8)	119 (100.0)	94 (79.0)	119 (100.0)	74 (62.2)	403 (99.8)	383 (94.8)
SBP > 130 mmHg or DBP > 85 mmHg	73 (62.4)	58 (49.6)	73 (61.3)	70 (58.8)	72 (60.5)	57 (47.9)	264 (65.3)	276 (68.3)
Triglycerides > 150 mg/dL	61 (52.1)	44 (37.6)	64 (53.8)	38 (31.9)	56 (47.1)	41 (34.5)	203 (50.2)	186 (46.0)
HDL < 40 mg/dL (M), < 50 mg/dL (F)	64 (54.7)	53 (45.3)	80 (67.2)	53 (44.5)	74 (62.2)	54 (45.4)	230 (56.9)	205 (50.7)

Data are n (%) at baseline and at the primary endpoint of 52 weeks (SURPASS-4) in patients on-treatment compliant to study drug (patients taking ≥ 75% of assigned doses). Percentage is calculated based on each subgroup value for each treatment group

DBP: diastolic blood pressure; F: female; FSG: fasting serum glucose; HbA1c: glycated hemoglobin; HDL: high-density lipoprotein; iDeg: insulin degludec; iGlar: insulin glargine; M: male; MET: metformin; n: number of patients in the specified category; SBP: systolic blood pressure; SGLT2i: sodium-glucose co-transporter 2 inhibitor; SU: sulfonylurea; WC: waist circumference

## Discussion

### Our findings in context

In the SURPASS clinical trial program, tirzepatide at all doses studied (5 mg, 10 mg, and 15 mg) demonstrated clinically relevant reductions in the prevalence of patients meeting the criteria for metabolic syndrome following 40 or 52 weeks of treatment. Reductions in the prevalence of metabolic syndrome with tirzepatide treatment were significantly greater versus active comparators. The magnitude of the effect was consistent among individual components of metabolic syndrome, with the exception of changes in HDL cholesterol. Greater body weight reduction with tirzepatide was associated with greater reduction in the prevalence of patients meeting the criteria for metabolic syndrome. A similar reduction in the prevalence of patients meeting the criteria for metabolic syndrome was observed despite patients on varying background medications across the T2D continuum.

To date, there is a lack of similar data with other non-surgical treatments for T2D. This may be because most other treatment options are associated with a lower decrease of weight. However, results of the current analysis align with a post hoc analysis of SURPASS-4, which demonstrated a significant dose-dependent reduction in the prevalence of patients meeting the criteria for metabolic syndrome from 83–88% at baseline to 51–60% at 52 weeks across groups compared to relatively no change in patients treated with insulin glargine [36]. Although there are data on metabolic improvement with semaglutide at doses of 0.5 mg and 1 mg in patients with T2D [37], there are no publications evaluating the improvement of metabolic syndrome in this population. In a post hoc analysis of the STEP 5 trial, in adults without diabetes and with a BMI ≥ 30 kg/m^2^ or ≥ 27 kg/m^2^ with ≥ 1 weight-related comorbidity, a greater proportion of patients treated with once-weekly subcutaneous semaglutide 2.4 mg achieved remission of metabolic syndrome, and fewer developed incident metabolic syndrome, compared with placebo. These benefits were maintained over 2 years of semaglutide treatment [38]. Similar results were observed in STEP 1, with resolution of metabolic syndrome observed in 63% of participants following 68 weeks of treatment with semaglutide 2.4 mg [39].

In the current analysis, all five individual risk factors of metabolic syndrome were improved after tirzepatide treatment, particularly the hyperglycemia component and to a lesser extent HDL cholesterol. This suggests both a weight loss-independent effect of tirzepatide, mediated by GIP and GLP-1 receptor agonism as demonstrated by an increase in pancreatic beta-cell glucose sensitivity and enhancing insulin secretion, and other weight loss-dependent effects such as waist circumference. Therefore, tirzepatide treatment increased the percentage of patients within a normal range for cardiovascular risk factors.

### The association of body weight reduction on the prevalence of patients meeting the criteria for metabolic syndrome

The reduction in the prevalence of patients meeting the criteria for metabolic syndrome in patients receiving tirzepatide and its individual components was associated with reductions in body weight. In the SURPASS clinical trial program, 7–43% of patients achieved ≥ 15% body weight reductions with tirzepatide [23–27]. A recent meta-analysis also demonstrated significant reductions in waist circumference in patients on tirzepatide [40]. Clinical characteristics of those achieving this much body weight loss and predictors of ≥ 15% body weight reductions have been previously assessed, determining female sex as one of the strongest predictors [41]. In the present analysis, despite of differences in baseline values, no meaningful sex differences in the magnitude of reduction of patients meeting either the overall criteria for metabolic syndromes or specifically the criterion of waist circumference could be determined (Additional file 1: Figures S1 and S2). The baseline prevalence of individuals meeting criteria of metabolic syndrome were somewhat larger in women and seems to be driven mainly by the waist circumference criteria. Moreover, the reduction of the prevalence of patients meeting the criteria for metabolic syndrome was not dependent on background SGLT2i or background use of sulfonylurea.

Modest weight loss of 5–10% has traditionally been a realistic goal for both preventing T2D and improving glycemic and metabolic control in people with T2D [41–43]. In the Diabetes Remission Clinical Trial (DiRECT), nearly half of patients with T2D of up to 6 years’ duration achieved remission, defined as HbA1c < 6.5% (< 48 mmol/mol) after at least 2 months off all antidiabetic medications, at 1 year following a structured weight-management program [44]. Furthermore, patients with higher weight loss were more likely to achieve HbA1c < 6.5%. At the 2-year follow-up, 17 (11%) of 149 patients achieved ≥ 15 kg weight loss, and of these, 70% achieved HbA1c < 6.5%, compared to 29% of patients who maintained ≥ 5 kg to < 10 kg weight loss [45]. The reduction in the prevalence of patients meeting the criteria for metabolic syndrome observed with tirzepatide treatment in this SURPASS clinical trial program, and in particular in those with substantial weight loss, is within a similar range that has been previously reported with surgical intervention [46]. This is notable considering that weight reduction with surgery is of a greater magnitude. Furthermore, both treatments have weight loss-independent benefits on certain aspects of the metabolic syndrome, such as glycemia [46].

The benefits of weight loss go beyond improving glycemic control, including reducing cardiovascular risk factors, and improving health-related quality of life and common obesity-related comorbidities of T2D, such as osteoarthritis and sleep apnea [47]. At the 1-year follow-up of the Look AHEAD randomized clinical trial, patients in the intensive lifestyle intervention group had greater improvements in HbA1c levels and in all cardiovascular risk factors, except for low-density lipoprotein cholesterol levels [17]. However, at a median follow-up of nearly 10 years, intensive lifestyle intervention did not decrease cardiovascular morbidity and mortality, as compared to a control program of diabetes support and education [17]. Furthermore, greater reductions in body weight are associated with greater improvements in blood pressure, glycemic control, and lipids [48]. Look AHEAD post hoc analyses showed that body weight reductions of 10% or increasing fitness by two metabolic equivalents in the first year was associated with an approximate 20% reduction in risk of cardiovascular disease, compared to no associations observed with small or moderate weight loss [18]. In the SELECT cardiovascular outcomes trial, treatment with once-weekly semaglutide 2.4 mg versus placebo resulted in superior reductions in MACE by 20% in adults living with overweight or obesity and established cardiovascular disease with no prior history of diabetes [49].

The results of this post hoc analysis align with tirzepatide mechanisms of action, such as the the improvement in insulin sensitivity. In Phase 2 and 3 trials, tirzepatide significantly reduced markers of insulin resistance (HOMA2-IR), and this finding was confirmed in a phase 1 mechanism of action study [29–31, 52]. This improvement can be explained in part by weight loss, since other mechanisms could be mediated, like the GIP activation in adipose tissue [29–31, 50–52]. Tirzepatide improved insulin sensitivity in people with T2D, with greater effects than semaglutide 1 mg, in a context of 11.2 kg loss with tirzepatide and 6.9 kg loss with semaglutide. [24, 31, 53]. In a post hoc analysis, tirzepatide 15 mg showed greater improvement in insulin sensitivity per unit weight loss than semaglutide 1 mg [54]. This aligns with the improvements observed in the current study, which demonstrated differences in the prevalence of patients meeting the criteria for metabolic syndrome between all tirzepatide doses and semaglutide.

The reduction in the proportion of patients with hypertension following tirzepatide treatment demonstrated in the present analysis may be, in part, due to weight loss. In the SURPASS studies, the reduction of the systolic blood pressure was primarily mediated through weight loss [40, 54, 55].

Tirzepatide treatment has reduced hypertriglyceridemia, an important component of metabolic syndrome. In the SURPASS-2 study, treatment with tirzepatide resulted in greater reductions in serum triglyceride concentration compared to selective GLP-1 receptor agonist semaglutide 1 mg [24]. Furthermore, the triglyceride-lowering effect of tirzepatide appears to be additive to the effect of fibrates [55, 56]. In a preclinical model, selective GIP agonist administration resulted in marked improvement of lipoprotein profile, suggesting that the GIP component may be an important contributor to metabolic improvements of GIP and GLP-1 receptor agonists, like tirzepatide [57]. The GIP agonist component of tirzepatide may contribute to the findings in most dimensions of metabolic syndrome. Apart from the favorable effect on lipoproteins, long-acting GIP agonists resulted in weight loss and, when combined with GLP-1 receptor agonists, contributed to better glycemic control [55].

Overall, our results align with other post hoc analyses with tirzepatide, which showed a greater clinical benefit, greater improvements in HbA1c and other cardiometabolic measures, with greater weight loss [41, 54].

### Limitations and conclusions

This analysis has limitations. This was a post hoc analysis that was exploratory in nature. Dose adjustments and indications for antihypertensive and lipid-modifying medications were not systematically collected to factor them into the assessment of metabolic syndrome status. Furthermore, reductions in body weight or HbA1c with tirzepatide did not plateau at Week 40/52 and therefore longer-term studies are needed to further elucidate their association on the prevalence of patients meeting the criteria for metabolic syndrome. Consequently, the true impact of tirzepatide on metabolic syndrome may not be fully be explained in this post hoc analysis. Interpretation of these results must consider that the metabolic syndrome criteria have been applied to a population with a diagnosis of diabetes mellitus and on antidiabetic treatment (except SURPASS-1) and many participants received treatment for hypertension or hypercholesterolemia. Furthermore, the predictive value of meeting metabolic syndrome criteria for cardiovascular risk and events may differ in patients with or without diabetes. However, in patients with T2D, metabolic syndrome has shown to predict atherosclerosis, is an independent predictor of cardiovascular disease, is an indicator of microvascular and macrovascular complications and increased risk of adverse cardio-renal outcomes [58–61]. Moreover, the present post hoc analysis did not include separate thresholds for waist circumference in Asians. While Asians, in particular South Asians, may be more susceptible to develop metabolic syndrome at waist circumferences below NCEP ATP III cutoff, the number of Asians included in this analysis was low and therefore would not have impacted outcome.

In conclusion, treatment with tirzepatide resulted in clinically relevant reductions in the prevalence of patients meeting the criteria for metabolic syndrome across the SURPASS clinical trial program. Greater body weight reduction with tirzepatide was associated with greater reduction in the prevalence of patients meeting the criteria for metabolic syndrome. Reducing the prevalence of individual components of metabolic syndrome may reduce the risk of cardiovascular events. The effect of tirzepatide treatment on cardiovascular risk factors and cardiovascular outcomes is being evaluated in the ongoing phase 3 study SURPASS-CVOT (NCT04255433).

### Supplementary Information

**Additional file 1.**

## Data Availability

Lilly provides access to all individual patient data collected during the trial, after anonymization, with the exception of pharmacokinetic or genetic data. Data are available to request 6 months after the indication studied has been approved in the US and EU and after primary publication acceptance, whichever is later. No expiration date of data requests is currently set once data are made available. Access is provided after a proposal has been approved by an independent review committee identified for this purpose and after receipt of a signed data sharing agreement. Data and documents, including the study protocol, statistical analysis plan, clinical study report, blank or annotated case report forms, will be provided in a secure data sharing environment. For details on submitting a request, see the instructions provided at www.vivli.org.

## Abbreviations

AHA/NHLBI: American Heart Association and the National Heart, Lung, and Blood Institute
BMI: Body mass index
CAD: Coronary artery disease
DBP: Diastolic blood pressure
F: Female
FSG: Fasting serum glucose
HbA1c: Glycated hemoglobin
HDL: High-density lipoprotein
iDeg: Insulin degludec
iGlar: Insulin glargine
GIP: Glucose-dependent insulinotropic polypeptide
GLP-1: Glucagon-like peptide-1
M: Male
MACE: Major adverse cardiovascular events
MET: Metformin
n: Number of patients in the specified category
PBO: Placebo
SBP: Systolic blood pressure
SEMA: Semaglutide
SGLT2i: Sodium-glucose co-transporter 2 inhibitor
SU: Sulfonylurea
T2D: Type 2 diabetes
US: United States
VLDL: Very-low-density lipoprotein
WC: Waist circumference

## References

[1] Reaven GM (1988). Banting Lecture. Role of insulin resistance in human disease. Diabetes.

[2] NCD Risk Factor Collaboration (NCD-RisC). Trends in adult body-mass index in 200 countries from 1975 to 2014: a pooled analysis of 1698 population-based measurement studies with 19.2 million participants. Lancet. 2016;387(10026):1377–96. 10.1016/S0140-6736(16)30054-X PMC7615134 27115820

[3] National Center for Health Statistics (US). Health, United States, 2015: With Special Feature on Racial and Ethnic Health Disparities. Hyattsville (MD): National Center for Health Statistics (US); 2016. Report No.: 2016–1232. 27308685

[4] Ford ES (2005). Risks for all-cause mortality, cardiovascular disease, and diabetes associated with the metabolic syndrome: a summary of the evidence. Diabetes Care.

[5] McNeill AM, Rosamond WD, Girman CJ, Golden SH, Schmidt MI, East HE, Ballantyne CM, Heiss G (2005). The metabolic syndrome and 11-year risk of incident cardiovascular disease in the atherosclerosis risk in communities study. Diabetes Care.

[6] Isomaa B, Almgren P, Tuomi T, Forsén B, Lahti K, Nissén M, Taskinen MR, Groop L (2001). Cardiovascular morbidity and mortality associated with the metabolic syndrome. Diabetes Care.

[7] Wilding JPH, Jacob S (2021). Cardiovascular outcome trials in obesity: A review. Obes Rev.

[8] Yu E, Ley SH, Manson JE, Willett W, Satija A, Hu FB, Stokes A (2017). Weight history and all-cause and cause-specific mortality in three prospective cohort studies. Ann Intern Med.

[9] Khan SS, Ning H, Wilkins JT, Allen N, Carnethon M, Berry JD, Sweis RN, Lloyd-Jones DM (2018). Association of body mass index with lifetime risk of cardiovascular disease and compression of morbidity. JAMA Cardiol.

[10] Lofgren IE, Herron KL, West KL, Zern TL, Brownbill RA, Ilich JZ, Koo SI, Fernandez ML (2005). Weight loss favorably modifies anthropometrics and reverses the metabolic syndrome in premenopausal women. J Am Coll Nutr.

[11] Poppitt SD, Keogh GF, Prentice AM, Williams DE, Sonnemans HM, Valk EE, Robinson E, Wareham NJ (2002). Long-term effects of ad libitum low-fat, high-carbohydrate diets on body weight and serum lipids in overweight subjects with metabolic syndrome. Am J Clin Nutr.

[12] Case CC, Jones PH, Nelson K, O'Brian Smith E, Ballantyne CM (2002). Impact of weight loss on the metabolic syndrome. Diabetes Obes Metab.

[13] Xydakis AM, Case CC, Jones PH, Hoogeveen RC, Liu MY, Smith EO, Nelson KW, Ballantyne CM (2004). Adiponectin, inflammation, and the expression of the metabolic syndrome in obese individuals: the impact of rapid weight loss through caloric restriction. J Clin Endocrinol Metab.

[14] Assali AR, Ganor A, Beigel Y, Shafer Z, Hershcovici T, Fainaru M (2001). Insulin resistance in obesity: body-weight or energy balance?. J Endocrinol.

[15] Nicklas BJ, Dennis KE, Berman DM, Sorkin J, Ryan AS, Goldberg AP (2003). Lifestyle intervention of hypocaloric dieting and walking reduces abdominal obesity and improves coronary heart disease risk factors in obese, postmenopausal, African-American and Caucasian women. J Gerontol A Biol Sci Med Sci.

[16] Einhorn D, Reaven GM, Cobin RH, Ford E, Ganda OP, Handelsman Y, Hellman R, Jellinger PS, Kendall D, Krauss RM, Neufeld ND, Petak SM, Rodbard HW, Seibel JA, Smith DA, Wilson PW (2003). American College of Endocrinology position statement on the insulin resistance syndrome. Endocr Pract.

[17] Wing RR, Bolin P, Brancati FL, Bray GA, Clark JM, Coday M, Crow RS, Curtis JM, Egan CM, Espeland MA, Evans M, Foreyt JP, Ghazarian S, Gregg EW, Barbara H, Helen PH, James OH, Horton ES, Hubbard VS, Jakicic JM, Jeffery RW, Johnson KC, Kahn SE, Kitabchi AE, Knowler WC, Lewis CE, Maschak-Carey BJ, Montez MG, Murillo A, Nathan DM, Patricio J, Peters A, Pi-Sunyer X, Pownall H, Reboussin D, Regensteiner JG, Rickman AD, Ryan DH, Safford M, Wadden TA, Wagenknecht LE, West DS, Williamson DF, Yanovski SZ (2013). Cardiovascular effects of intensive lifestyle intervention in type 2 diabetes. N Engl J Med.

[18] Gregg EW, Jakicic JM, Blackburn G, Bloomquist P, Bray GA, Clark JM, Coday M, Curtis JM, Egan C, Evans M, Foreyt J, Foster G, Hazuda HP, Hill JO, Horton ES, Hubbard VS, Jeffery RW, Johnson KC, Kitabchi AE, Knowler WC, Kriska A, Lang W, Lewis CE, Montez MG, Nathan DM, Neiberg RH, Patricio J, Peters A, Pi-Sunyer X, Pownall H, Redmon B, Regensteiner J, Rejeski J, Ribisl PM, Safford M, Stewart K, Trence D, Wadden TA, Wing RR, Yanovski SZ (2016). Association of the magnitude of weight loss and changes in physical fitness with long-term cardiovascular disease outcomes in overweight or obese people with type 2 diabetes: a post-hoc analysis of the Look AHEAD randomised clinical trial. Lancet Diabetes Endocrinol.

[19] Schauer PR, Kashyap SR, Wolski K, Brethauer SA, Kirwan JP, Pothier CE, Thomas S, Abood B, Nissen SE, Bhatt DL (2012). Bariatric surgery versus intensive medical therapy in obese patients with diabetes. N Engl J Med.

[20] Schauer PR, Bhatt DL, Kirwan JP, Wolski K, Brethauer SA, Navaneethan SD, Aminian A, Pothier CE, Kim ES, Nissen SE, Kashyap SR (2014). Bariatric surgery versus intensive medical therapy for diabetes–3-year outcomes. N Engl J Med.

[21] Schauer PR, Bhatt DL, Kirwan JP, Wolski K, Aminian A, Brethauer SA, Navaneethan SD, Singh RP, Pothier CE, Nissen SE, Kashyap SR (2017). Bariatric surgery versus intensive medical therapy for diabetes - 5-year outcomes. N Engl J Med.

[22] Kashyap SR, Bhatt DL, Wolski K, Watanabe RM, Abdul-Ghani M, Abood B, Pothier CE, Brethauer S, Nissen S, Gupta M, Kirwan JP, Schauer PR (2013). Metabolic effects of bariatric surgery in patients with moderate obesity and type 2 diabetes: analysis of a randomized control trial comparing surgery with intensive medical treatment. Diabetes Care.

[23] Rosenstock J, Wysham C, Frías JP, Kaneko S, Lee CJ, Fernández Landó L, Mao H, Cui X, Karanikas CA, Thieu VT (2021). Efficacy and safety of a novel dual GIP and GLP-1 receptor agonist tirzepatide in patients with type 2 diabetes (SURPASS-1): a double-blind, randomised, phase 3 trial. Lancet.

[24] Frías JP, Davies MJ, Rosenstock J, Pérez Manghi FC, Fernández Landó L, Bergman BK, Liu B, Cui X, Brown K (2021). Tirzepatide versus semaglutide once weekly in patients with type 2 diabetes. N Engl J Med.

[25] Ludvik B, Giorgino F, Jódar E, Frias JP, Fernández Landó L, Brown K, Bray R, Rodríguez Á (2021). Once-weekly tirzepatide versus once-daily insulin degludec as add-on to metformin with or without SGLT2 inhibitors in patients with type 2 diabetes (SURPASS-3): a randomised, open-label, parallel-group, phase 3 trial. Lancet.

[26] Del Prato S, Kahn SE, Pavo I, Weerakkody GJ, Yang Z, Doupis J, Aizenberg D, Wynne AG, Riesmeyer JS, Heine RJ, Wiese RJ (2021). Tirzepatide versus insulin glargine in type 2 diabetes and increased cardiovascular risk (SURPASS-4): a randomised, open-label, parallel-group, multicentre, phase 3 trial. Lancet.

[27] Dahl D, Onishi Y, Norwood P, Huh R, Bray R, Patel H, Rodríguez Á (2022). Effect of subcutaneous tirzepatide vs placebo added to titrated insulin glargine on glycemic control in patients with type 2 diabetes: The SURPASS-5 randomized clinical trial. JAMA.

[28] Garvey WT, Frias JP, Jastreboff AM, le Roux CW, Sattar N, Aizenberg D, Mao H, Zhang S, Ahmad NN, Bunck MC, Benabbad I, Zhang XM (2023). Tirzepatide once weekly for the treatment of obesity in people with type 2 diabetes (SURMOUNT-2): a double-blind, randomised, multicentre, placebo-controlled, phase 3 trial. Lancet.

[29] Lee CJ, Mao H, Thieu VT, Landó LF, Thomas MK (2023). Tirzepatide as monotherapy improved markers of beta-cell function and insulin sensitivity in type 2 diabetes (SURPASS-1). J Endocr Soc..

[30] Heise T, Mari A, DeVries JH, Urva S, Li J, Pratt EJ, Coskun T, Thomas MK, Mather KJ, Haupt A, Milicevic Z (2022). Effects of subcutaneous tirzepatide versus placebo or semaglutide on pancreatic islet function and insulin sensitivity in adults with type 2 diabetes: a multicentre, randomised, double-blind, parallel-arm, phase 1 clinical trial. Lancet Diabetes Endocrinol.

[31] Thomas MK, Nikooienejad A, Bray R, Cui X, Wilson J, Duffin K, Milicevic Z, Haupt A, Robins DA (2021). Dual GIP and GLP-1 receptor agonist tirzepatide improves beta-cell function and insulin sensitivity in type 2 diabetes. J Clin Endocrinol Metab.

[32] Sattar N, McGuire DK, Pavo I, Weerakkody GJ, Nishiyama H, Wiese RJ, Zoungas S (2022). Tirzepatide cardiovascular event risk assessment: a pre-specified meta-analysis. Nat Med.

[33] Pirro V, Roth KD, Lin Y, Willency JA, Milligan PL, Wilson JM, Ruotolo G, Haupt A, Newgard CB, Duffin KL (2022). Effects of tirzepatide, a dual GIP and GLP-1 RA, on lipid and metabolite profiles in subjects with type 2 diabetes. J Clin Endocrinol Metab.

[34] Grundy SM, Cleeman JI, Daniels SR, Donato KA, Eckel RH, Franklin BA, Gordon DJ, Krauss RM, Savage PJ, Smith SC, Spertus JA, Costa F (2005). American Heart Association; National Heart, Lung, and Blood Institute: Diagnosis and management of the metabolic syndrome: An American Heart Association/National Heart, Lung, and Blood Institute Scientific statement. Circulation.

[35] Expert Panel on Detection, Evaluation, and Treatment of High Blood Cholesterol in Adults. Executive Summary of The Third Report of The National Cholesterol Education Program (NCEP) Expert Panel on Detection, Evaluation, and Treatment of High Blood Cholesterol in Adults (Adult Treatment Panel III). JAMA. 2001;285(19):2486–97. 10.1001/jama.285.19.2486 11368702

[36] Nicholls SJ, D’Alessio DA, Wiese RJ, Pavo I, Zeytinoglu M, Romera IC, Weerakkody GJ (2023). Compared to insulin glargine, tirzepatide reduces the prevalence of the metabolic syndrome in patients with type 2 diabetes and high cardiovascular risk: Post-hoc analysis of SURPASS-4. J Am Coll Cardiol.

[37] Linong Ji, Andrew Ahmann, Bo Ahrén, Matthew Capehorn, Ping Hu, Ildiko Lingvay, Wenyan Liu, Helena W. Rodbard, Zewei Shen, Christopher H. Sorli; 746-P: Semaglutide Increases the Proportion of People with T2D Achieving a Metabolic Composite Endpoint. Diabetes. 2023;72(Suppl):746–P.

[38] Batterham RL, Holst-Hansen T, Kandler K, Rigas G, Garvey WT (2022). Impact of once-weekly subcutaneous semaglutide 2.4 mg on metabolic syndrome in the 2-year, randomised controlled STEP 5 trial. Diabetologia.

[39] le Roux CW, Davies M, Frias JP, Jensen C, Nørkjær Laursen P, Lingvay I, Machineni S, Varbo A, Wilding JPH, Perreault L (2021). Once-weekly semaglutide 2.4 mg improved metabolic syndrome in adults with overweight or obesity: Post-hoc analysis of the STEP 1 trial. Obes Facts.

[40] Yu D, Shen S, Zhang J, Wang Q (2023). Effect of the dual glucose-dependent insulinotropic peptide/gulcagon-like peptide 1 receptor agonist tirzepatide on lipid profile and waist circumference: a systematic review and meta-analysis. Clin Ther.

[41] Małecki MT, Batterham RL, Sattar N, Levine JA, Rodríguez Á, Bergman BK, Wang H, Ghimpeteanu G, Lee CJ (2023). Predictors of ≥15% weight reduction and associated changes in cardiometabolic risk factors with tirzepatide in adults with type 2 diabetes in SURPASS 1–4. Diabetes Care.

[42] Lingvay I, Sumithran P, Cohen RV, le Roux CW (2022). Obesity management as a primary treatment goal for type 2 diabetes: time to reframe the conversation. Lancet.

[43] Davies MJ, Aroda VR, Collins BS, Gabbay RA, Green J, Maruthur NM, Rosas SE, Del Prato S, Mathieu C, Mingrone G, Rossing P, Tankova T, Tsapas A, Buse JB (2022). Management of hyperglycemia in type 2 diabetes, 2022. A consensus report by the American Diabetes Association (ADA) and the European Association for the Study of Diabetes (EASD). Diabetes Care.

[44] Lean ME, Leslie WS, Barnes AC, Brosnahan N, Thom G, McCombie L, Peters C, Zhyzhneuskaya S, Al-Mrabeh A, Hollingsworth KG, Rodrigues AM, Rehackova L, Adamson AJ, Sniehotta FF, Mathers JC, Ross HM, McIlvenna Y, Stefanetti R, Trenell M, Welsh P, Kean S, Ford I, McConnachie A, Sattar N, Taylor R (2018). Primary care-led weight management for remission of type 2 diabetes (DiRECT): an open-label, cluster-randomised trial. Lancet.

[45] Lean MEJ, Leslie WS, Barnes AC, Brosnahan N, Thom G, McCombie L, Peters C, Zhyzhneuskaya S, Al-Mrabeh A, Hollingsworth KG, Rodrigues AM, Rehackova L, Adamson AJ, Sniehotta FF, Mathers JC, Ross HM, McIlvenna Y, Welsh P, Kean S, Ford I, McConnachie A, Messow CM, Sattar N, Taylor R (2019). Durability of a primary care-led weight-management intervention for remission of type 2 diabetes: 2-year results of the DiRECT open-label, cluster-randomised trial. Lancet Diabetes Endocrinol.

[46] Ooi GJ, Doyle L, Tie T, Wentworth JM, Laurie C, Earnest A, Cowley MA, Sikaris K, le Roux CW, Burton PR, O'Brien PE, Brown WA (2017). Weight loss after laparoscopic adjustable gastric band and resolution of the metabolic syndrome and its components. Int J Obes (Lond).

[47] Clinical guidelines on the identification (1998). evaluation, and treatment of overweight and obesity in adults–The Evidence report. National Institutes of Health Obes Res.

[48] Wing RR, Lang W, Wadden TA, Safford M, Knowler WC, Bertoni AG, Hill JO, Brancati FL, Peters A, Wagenknecht L (2011). Benefits of modest weight loss in improving cardiovascular risk factors in overweight and obese individuals with type 2 diabetes. Diabetes Care.

[49] Lincoff AM, Brown-Frandsen K, Colhoun HM, Deanfield J, Emerson SS, Esbjerg S, Hardt-Lindberg S, Hovingh GK, Kahn SE, Kushner RF, Lingvay I, Oral TK, Michelsen MM, Plutzky J, Tornøe CW, Ryan DH (2023). Semaglutide and Cardiovascular Outcomes in Obesity without Diabetes. N Engl J Med.

[50] Coskun T, Sloop KW, Loghin C, Alsina-Fernandez J, Urva S, Bokvist KB, Cui X, Briere DA, Cabrera O, Roell WC, Kuchibhotla U, Moyers JS, Benson CT, Gimeno RE, D'Alessio DA, Haupt A (2018). LY3298176, a novel dual GIP and GLP-1 receptor agonist for the treatment of type 2 diabetes mellitus: From discovery to clinical proof of concept. Mol Metab.

[51] Heise T, DeVries JH, Urva S, Li J, Pratt EJ, Thomas MK, Mather KJ, Karanikas CA, Dunn J, Haupt A, Milicevic Z, Coskun T (2023). Tirzepatide reduces appetite, energy intake, and fat mass in people with type 2 diabetes. Diabetes Care.

[52] Brown K, Fernandez LL, Bergman B, Thomas MK, Liu B, Lee C (2022). Tirzepatide improved markers of islet-cell function (fasting glucagon and HOMA2-B) and insulin sensitivity (fasting insulin and HOMA2-IR) Compared with semaglutide in people with type 2 diabetes. Diabetes.

[53] Mather KJ, Mari A, Li J, Heise T, De Vries JH, Urva S, Coskun T, Milicevic Z, Haupt A, Thomas MK (2022). 714-P: Greater improvement in insulin sensitivity per unit weight loss with tirzepatide compared with selective GLP-1 receptor agonism. Diabetes.

[54] Lingvay I, Mosenzon O, Brown K, Cui X, O'Neill C, Fernández Landó L, Patel H (2023). Systolic blood pressure reduction with tirzepatide in patients with type 2 diabetes: insights from SURPASS clinical program. Cardiovasc Diabetol.

[55] Várkonyi TT, Pósa A, Pávó N, Pavo I (2023). Perspectives on weight control in diabetes - Tirzepatide. Diabetes Res Clin Pract.

[56] Várkonyi T, Del Prato S, Pavo I, Nicolay C, Wiese RJ, Kahn SE (2022). Tirzepatide reduces serum triglyceride concentrations irrespective of concomitant fibrate use in SURPASS-4 participants with type 2 diabetes at high cardiovascular risk. Diabetologia.

[57] Sachs S, Götz A, Finan B, Feuchtinger A, DiMarchi RD, Döring Y, Weber C, Tschöp MH, Müller TD, Hofmann SM (2023). GIP receptor agonism improves dyslipidemia and atherosclerosis independently of body weight loss in preclinical mouse model for cardio-metabolic disease. Cardiovasc Diabetol.

[58] Ferreira JP, Verma S, Fitchett D, Ofstad AP, Lauer S, Zwiener I, George J, Wanner C, Zinman B, Inzucchi SE (2020). Metabolic syndrome in patients with type 2 diabetes and atherosclerotic cardiovascular disease: a post hoc analyses of the EMPA-REG OUTCOME trial. Cardiovasc Diabetol.

[59] Bonadonna R, Cucinotta D, Fedele D, Riccardi G, Tiengo A (2006). The metabolic syndrome is a risk indicator of microvascular and macrovascular complications in diabetes: results from Metascreen, a multicenter diabetes clinic-based survey. Diabetes Care.

[60] Alexander CM, Landsman PB, Teutsch SM, Haffner SM (2003). Third National Health and Nutrition Examination Survey (NHANES III); National Cholesterol Education Program (NCEP). NCEP-defined metabolic syndrome, diabetes, and prevalence of coronary heart disease among NHANES III participants age 50 years and older. Diabetes.

[61] Essafi MA, Bouabdellaoui L, Aynaou H, Salhi H, El Ouahabi H (2022). Metabolic syndrome in patients with diabetes mellitus. Cureus..

