# Supplementary material for: Reduction of prevalence of patients meeting the criteria for metabolic syndrome with tirzepatide: a post hoc analysis from the SURPASS Clinical Trial Program
Source: Cardiovasc Diabetol. 2024 Feb 10;23:63. doi: 10.1186/s12933-024-02147-9 (PMC10859014; doi:10.1186/s12933-024-02147-9)
Supplement: Supplementary file 1 — Additional file 1. [file 12933_2024_2147_MOESM1_ESM.docx]

**ONLINE-ONLY SUPPLEMENTAL APPENDIX**

**Table S1. Criteria for metabolic syndrome**

| **Clinical characteristic** | **Categorical cutoffs are based on NCEP ATP III (2005 revision)^1,2^** |
| --- | --- |
| **Obesity** | waist circumference >102 cm (40 inches) in males, >89 cm (35 inches) in females |
| **Hyperglycemia** | fasting serum glucose ≥100 mg/dL or HbA1c ≥5.7% |
| **Hypertension** | systolic blood pressure >130 mmHg or diastolic blood pressure >85 mmHg |
| **Dyslipidemia** | triglycerides >150 mg/dL and/or high-density lipoprotein cholesterol <40 mg/dL in males, <50 mg/dL in females |

Any 3 of the 5 categorical cutoffs constitute diagnosis of metabolic syndrome. 1. Grundy et al. Circulation. 2005;112(17):2735-52. 2. Expert Panel on Detection, Evaluation, and Treatment of High Blood Cholesterol in Adults. JAMA. 2001;285(19):2486-97

**Table S2. Prevalence of patients meeting the criteria for metabolic syndrome in tirzepatide-treated patients by weight loss category (<15% or ≥15%)**

| **Metabolic Syndrome Risk Factors** | **Weight Loss <15%** | | **Weight Loss ≥15%)** | |
| --- | --- | --- | --- | --- |
|  | **Baseline** | **Primary Endpoint** | **Baseline** | **Primary Endpoint** |
| **SURPASS-1 monotherapy, N** | **242** | | **59** | |
| **≥3 Risk Factors** | 171 (70.7) | 126 (52.1) | 45 (76.3) | 17 (28.8) |
| **WC >102 cm (M), >89 cm (F)** | 161 (66.5) | 129 (53.3) | 43 (72.9) | 27 (45.8) |
| **FSG ≥100 mg/dL or HbA1c ≥5.7%** | 242 (100.0) | 197 (81.4) | 59 (100.0) | 30 (50.8) |
| **SBP >130 mmHg or DBP >85 mmHg** | 112 (46.3) | 84 (34.7) | 27 (45.8) | 11 (18.6) |
| **Triglycerides >150 mg/dL** | 123 (50.8) | 84 (34.7) | 26 (44.1) | 7 (11.9) |
| **HDL <40 mg/dL (M), <50 mg/dL (F)** | 131 (54.1) | 118 (48.8) | 37 (62.7) | 32 (54.2) |
| **SURPASS-2 add-on to MET vs SEMA 1 mg, N** | **865** | | **326** | |
| **≥3 Risk Factors** | 727 (84.0) | 504 (58.3) | 278 (85.3) | 83 (25.5) |
| **WC >102 cm (M), >89 cm (F)** | 715 (82.7) | 595 (68.8) | 282 (86.5) | 155 (47.5) |
| **FSG ≥100 mg/dL or HbA1c ≥5.7%** | 865 (100.0) | 708 (81.8) | 326 (100.0) | 135 (41.4) |
| **SBP >130 mmHg or DBP >85 mmHg** | 501 (57.9) | 370 (42.8) | 179 (54.9) | 82 (25.2) |
| **Triglycerides >150 mg/dL** | 485 (56.1) | 343 (39.7) | 162 (49.7) | 68 (20.9) |
| **HDL <40 mg/dL (M), <50 mg/dL (F)** | 485 (56.1) | 387 (44.7) | 190 (58.3) | 142 (43.6) |
| **SURPASS-3 add-on to MET ± SGLT2i vs iDeg, N** | **644** | | **247** | |
| **≥3 Risk Factors** | 538 (83.5) | 394 (61.2) | 205 (83.0) | 74 (30.0) |
| **WC >102 cm (M), >89 cm (F)** | 537 (83.4) | 438 (68.0) | 218 (88.3) | 117 (47.4) |
| **FSG ≥100 mg/dL or HbA1c ≥5.7%** | 644 (100.0) | 566 (87.9) | 247 (100.0) | 134 (54.3) |
| **SBP >130 mmHg or DBP >85 mmHg** | 399 (62.0) | 284 (44.1) | 139 (56.3) | 68 (27.5) |
| **Triglycerides >150 mg/dL** | 348 (54.0) | 265 (41.1) | 136 (55.1) | 47 (19.0) |
| **HDL <40 mg/dL (M), <50 mg/dL (F)** | 342 (53.1) | 282 (43.8) | 145 (58.7) | 96 (38.9) |
| **SURPASS-4 ± MET ± SGLT2i ± SU vs iGlar, N** | **616** | | **207** | |
| **≥3 Risk Factors** | 520 (84.4) | 388 (63.0) | 186 (89.9) | 75 (36.2) |
| **WC >102 cm (M), >89 cm (F)** | 494 (80.2) | 394 (64.0) | 181 (87.4) | 101 (48.8) |
| **FSG ≥100 mg/dL or HbA1c ≥5.7%** | 616 (100.0) | 538 (87.3) | 207 (100.0) | 109 (52.7) |
| **SBP >130 mmHg or DBP >85 mmHg** | 403 (65.4) | 324 (52.6) | 125 (60.4) | 103 (49.8) |
| **Triglycerides >150 mg/dL** | 346 (56.2) | 243 (39.4) | 105 (50.7) | 53 (25.6) |
| **HDL <40 mg/dL (M), <50 mg/dL (F)** | 365 (59.3) | 302 (49.0) | 141 (68.1) | 82 (39.6) |
| **SURPASS-5 add-on to insulin glargine ± MET vs PBO, N** | **246** | | **62** | |
| **≥3 Risk Factors** | 195 (79.3) | 144 (58.5) | 48 (77.4) | 15 (24.2) |
| **WC >102 cm (M), >89 cm (F)** | 211 (85.8) | 194 (78.9) | 49 (79.0) | 30 (48.4) |
| **FSG ≥100 mg/dL or HbA1c ≥5.7%** | 246 (100.0) | 189 (76.8) | 62 (100.0) | 17 (27.4) |
| **SBP >130 mmHg or DBP >85 mmHg** | 175 (71.1) | 127 (51.6) | 45 (72.6) | 18 (29.0) |
| **Triglycerides >150 mg/dL** | 108 (43.9) | 71 (28.9) | 29 (46.8) | 10 (16.1) |
| **HDL <40 mg/dL (M), <50 mg/dL (F)** | 117 (47.6) | 105 (42.7) | 24 (38.7) | 21 (33.9) |

Data are n (%) at baseline and at the primary endpoint of 40 weeks (SURPASS-1, SURPASS-2 and SURPASS-5) or 52 weeks (SURPASS-3, SURPASS-4) in patients on-treatment compliant to study drug (patients taking ≥75% of assigned doses). Abbreviations: DBP=diastolic blood pressure; F=female; FSG=fasting serum glucose; HbA1c=glycated hemoglobin; HDL=high-density lipoprotein; iDeg=insulin degludec; iGlar=insulin glargine; M=male; MET=metformin; n=number of patients in the specified category; PBO=placebo; SBP=systolic blood pressure; SEMA=semaglutide; SGLT2i=sodium-glucose co-transporter 2 inhibitor; SU=sulfonylurea; WC=waist circumference.

**Table S3. Prevalence of patients meeting the criteria for metabolic syndrome by SGLT2i status (yes, no)**

| **Metabolic Syndrome Risk Factors** | **Tirzepatide 5 mg** | | **Tirzepatide 10 mg** | | **Tirzepatide 15 mg** | | **Comparator** | |
| --- | --- | --- | --- | --- | --- | --- | --- | --- |
|  | **Baseline** | **Primary Endpoint** | **Baseline** | **Primary Endpoint** | **Baseline** | **Primary Endpoint** | **Baseline** | **Primary Endpoint** |
| **SURPASS-3 add-on to MET ± SGLT2i vs iDeg, N** | | | | | | | | |
| **SGLT2i, yes** | **99** | | **104** | | **97** | | **108** | |
| **≥3 Risk Factors** | 83 (83.8) | 61 (61.6) | 84 (80.8) | 45 (43.3) | 77 (79.4) | 50 (51.5) | 81 (75.0) | 85 (78.7) |
| **WC >102 cm (M), >89 cm (F)** | 85 (85.9) | 69 (69.7) | 82 (78.8) | 53 (51.0) | 83 (85.6) | 55 (56.7) | 88 (81.5) | 88 (81.5) |
| **FSG ≥100 mg/dL or HbA1c ≥5.7%** | 99 (100.0) | 90 (90.9) | 104 (100.0) | 80 (76.9) | 97 (100.0) | 81 (83.5) | 108 (100.0) | 105 (97.2) |
| **SBP >130 mmHg or DBP >85 mmHg** | 57 (57.6) | 40 (40.4 | 59 (56.7) | 38 (36.5) | 54 (55.7) | 33 (34.0) | 66 (61.1) | 70 (64.8) |
| **Triglycerides >150 mg/dL** | 62 (62.6) | 42 (42.4) | 59 (56.7) | 30 (28.8) | 55 (56.7) | 35 (36.1) | 60 (55.6) | 43 (39.8) |
| **HDL <40 mg/dL (M), <50 mg/dL (F)** | 50 (50.5) | 43 (43.4) | 53 (51.0) | 37 (35.6) | 44 (45.4) | 36 (37.1) | 49 (45.4) | 43 (39.8) |
| **SGLT2i, no** | **209** | | **187** | | **195** | | **205** | |
| **≥3 Risk Factors** | 162 (77.5) | 123 (58.9) | 165 (88.2) | 94 (50.3) | 172 (88.2) | 95 (48.7) | 161 (78.5) | 163 (79.5) |
| **WC >102 cm (M), >89 cm (F)** | 170 (81.3 | 143 (68.4) | 166 (88.8) | 117 (62.6) | 169 (86.7) | 118 (60.5) | 165 (80.5) | 175 (85.4) |
| **FSG ≥100 mg/dL or HbA1c ≥5.7%** | 209 (100.0) | 179 (85.6 | 187 (100.0) | 140 (74.9) | 195 (100.0) | 130 (66.7) | 205 (100.0) | 192 (93.7) |
| **SBP >130 mmHg or DBP >85 mmHg** | 128 (61.2) | 91 (43.5) | 114 (61.0) | 71 (38.0) | 126 (64.6) | 79 (40.5) | 135 (65.9) | 122 (59.5) |
| **Triglycerides >150 mg/dL** | 104 (49.8) | 78 (37.3) | 96 (51.3 | 61 (32.6) | 108 (55.4) | 66 (33.8) | 95 (46.3) | 86 (42.0) |
| **HDL <40 mg/dL (M), <50 mg/dL (F)** | 103 (49.3) | 92 (44.0) | 118 (63.1) | 81 (43.3) | 119 (61.0) | 89 (45.6) | 105 (51.2) | 107 (52.2) |
| **SURPASS-4 ± MET ± SGLT2i ± SU vs iGlar, N** | | | | | | | | |
| **SGLT2i, yes** | **69** | | **65** | | **71** | | **223** | |
| **≥3 Risk Factors** | 56 (81.2) | 40 (58.0) | 59 (90.8) | 40 (61.5) | 62 (87.3) | 34 (47.9) | 177 (79.4) | 180 (80.7) |
| **WC >102 cm (M), >89 cm (F)** | 55 (79.7) | 42 (60.9) | 58 (89.2) | 46 (70.8) | 61 (85.9) | 41 (57.7) | 164 (73.5) | 175 (78.5) |
| **FSG ≥100 mg/dL or HbA1c ≥5.7%** | 69 (100.0) | 58 (84.1) | 65 (100.0) | 56 (86.2) | 71 (100.0) | 52 (73.2) | 223 (100.0) | 215 (96.4) |
| **SBP >130 mmHg or DBP >85 mmHg** | 38 (55.1) | 32 (46.4) | 39 (60.0) | 34 (52.3) | 37 (52.1) | 28 (39.4) | 133 (59.6) | 145 (65.0) |
| **Triglycerides >150 mg/dL** | 36 (52.2) | 30 (43.5) | 37 (56.9) | 22 (33.8) | 41 (57.7) | 25 (35.2) | 116 (52.0) | 109 (48.9) |
| **HDL <40 mg/dL (M), <50 mg/dL (F)** | 40 (58.0) | 30 (43.5) | 44 (67.7) | 29 (44.6) | 45 (63.4) | 32 (45.1) | 123 (55.2) | 115 (51.6) |
| **SGLT2i, no** | **204** | | **211** | | **203** | | **647** | |
| **≥3 Risk Factors** | 171 (83.8) | 119 (58.3) | 183 (86.7) | 123 (58.3) | 175 (86.2) | 107 (52.7) | 550 (85.0) | 537 (83.0) |
| **WC >102 cm (M), >89 cm (F)** | 167 (81.9) | 132 (64.7) | 169 (80.1) | 117 (55.5) | 165 (81.3) | 117 (57.6) | 510 (78.8) | 534 (82.5) |
| **FSG ≥100 mg/dL or HbA1c ≥5.7%** | 204 (100.0) | 169 (82.8) | 211 (100.0) | 171 (81.0) | 203 (100.0) | 141 (69.5) | 646 (99.8) | 628 (97.1) |
| **SBP >130 mmHg or DBP >85 mmHg** | 131 (64.2) | 102 (50.0) | 141 (66.8) | 121 (57.3) | 142 (70.0) | 110 (54.2) | 427 (66.0) | 464 (71.7) |
| **Triglycerides >150 mg/dL** | 118 (57.8) | 82 (40.2) | 109 (51.7) | 70 (33.2) | 110 (54.2) | 67 (33.0) | 335 (51.8) | 307 (47.4) |
| **HDL <40 mg/dL (M), <50 mg/dL (F)** | 116 (56.9) | 100 (49.0) | 138 (65.4) | 101 (47.9) | 123 (60.6) | 92 (45.3) | 386 (59.7) | 351 (54.3) |

Data are n (%) at baseline and at the primary endpoint of 52 weeks (SURPASS-3, SURPASS-4) in patients on-treatment compliant to study drug (patients taking ≥75% of assigned doses). Percentage is calculated based on each subgroup value for each treatment group. Abbreviations: DBP=diastolic blood pressure; F=female; FSG=fasting serum glucose; HbA1c=glycated hemoglobin; HDL=high-density lipoprotein; iDeg=insulin degludec; iGlar=insulin glargine; M=male; MET=metformin; n=number of patients in the specified category; SBP=systolic blood pressure; SGLT2i=sodium-glucose co-transporter 2 inhibitor; SU=sulfonylurea; WC=waist circumference.

**FIGURE S1. Prevalence of patients meeting criteria for metabolic syndrome by gender (female, male)**


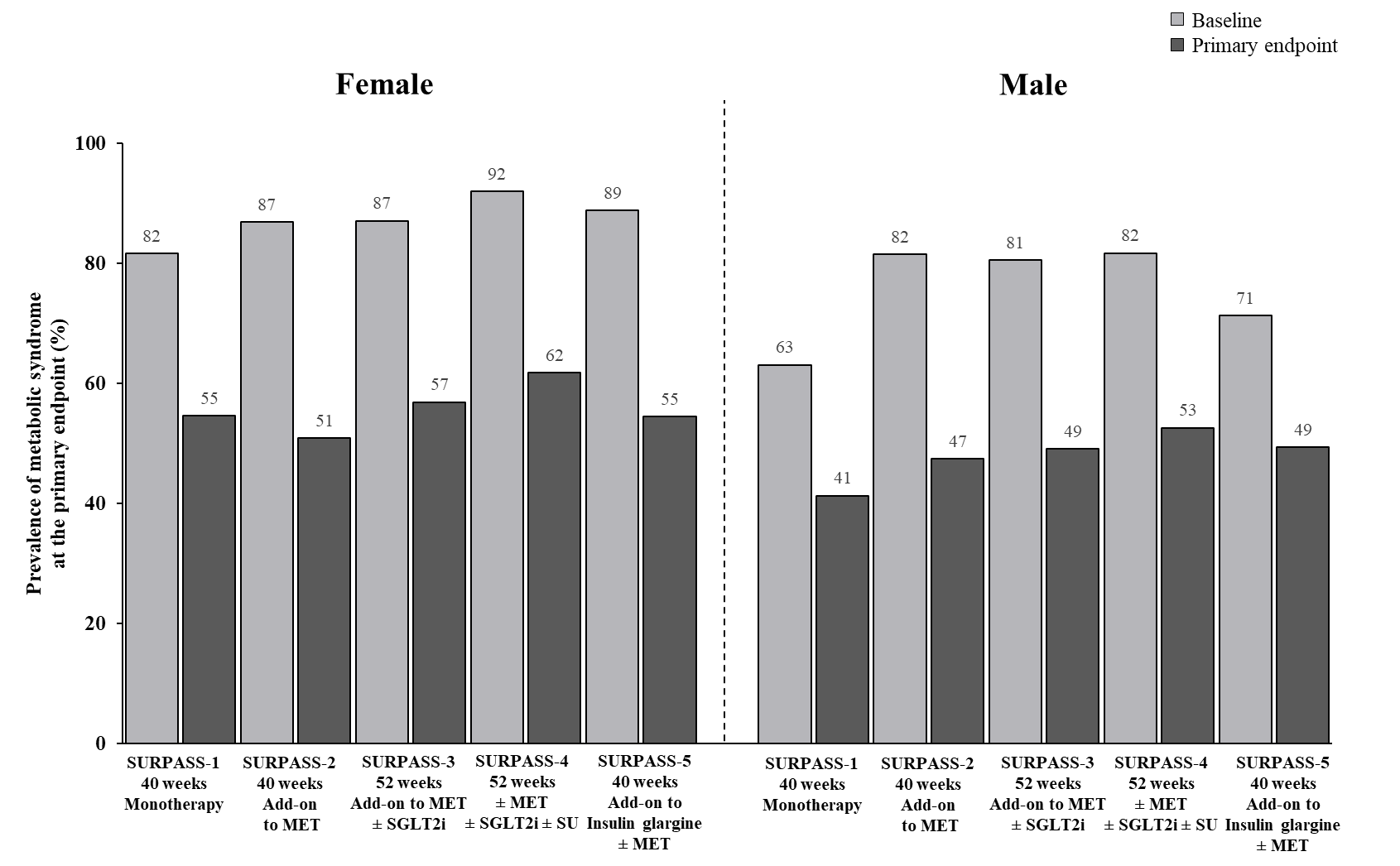


Data are proportion of tirzepatide-treated patients with at least 3 diagnostic criteria for metabolic syndrome at baseline and at the primary endpoint of 40/52 weeks by gender (female, male). Abbreviations: MET=metformin; SGLT2i=sodium-glucose co-transporter 2 inhibitor; SU=sulfonylurea.

**FIGURE S2. Prevalence of patients meeting the waist circumference component of metabolic syndrome by gender (female, male)**


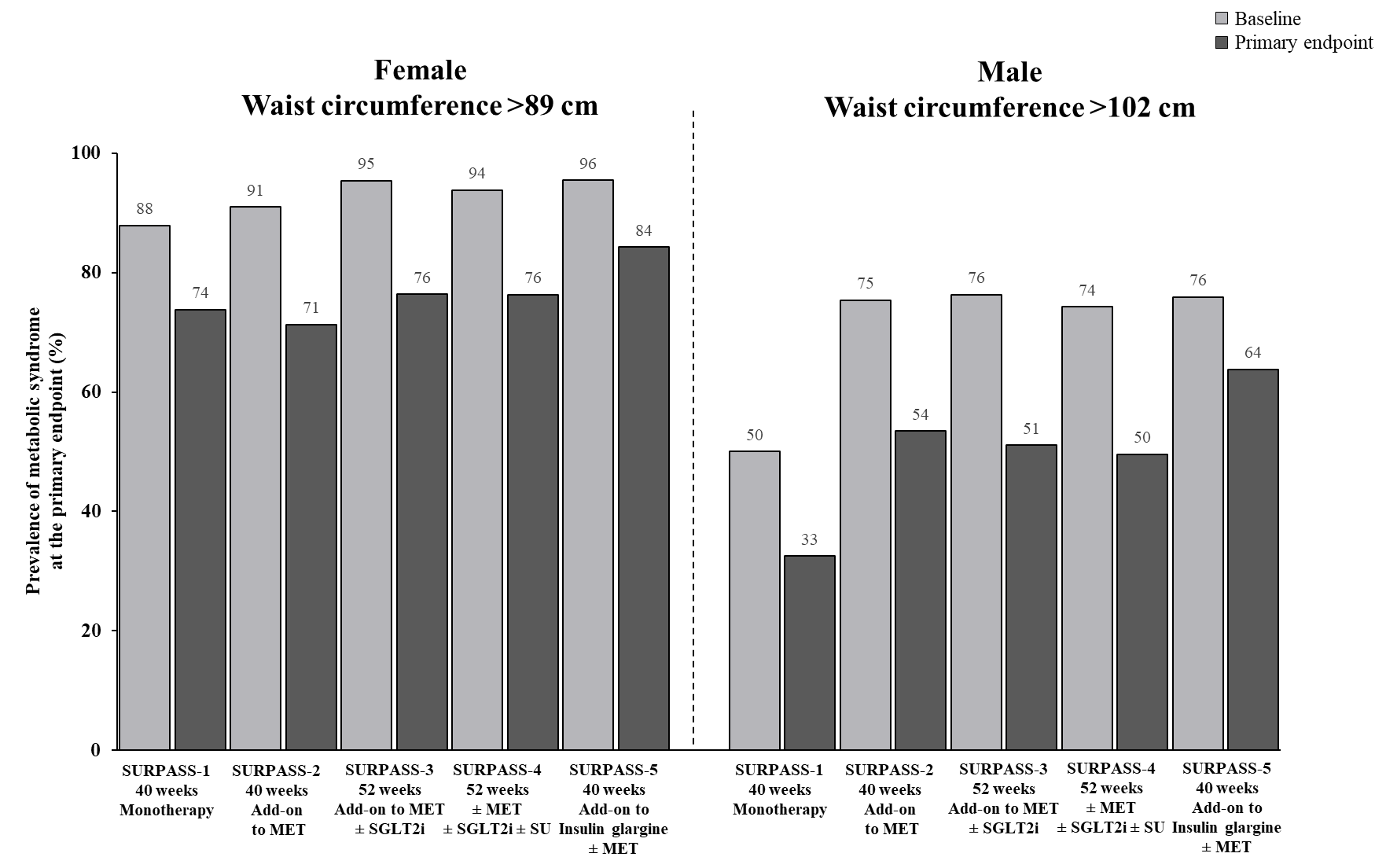


Data are proportion of tirzepatide-treated patients meeting the waist circumference component of metabolic syndrome at baseline and at the primary endpoint of 40/52 weeks by gender (female, male). Abbreviations: MET=metformin; SGLT2i=sodium-glucose co-transporter 2 inhibitor; SU=sulfonylurea.
